# In Vitro Antioxidant Activity and FTIR Characterization of High-Molecular Weight Melanoidin Fractions from Different Types of Cocoa Beans

**DOI:** 10.3390/antiox8110560

**Published:** 2019-11-15

**Authors:** Joanna Oracz, Dorota Zyzelewicz

**Affiliations:** Institute of Food Technology and Analysis, Faculty of Biotechnology and Food Sciences, Lodz University of Technology, 4/10 Stefanowskiego Street, 90-924 Lodz, Poland; dorota.zyzelewicz@p.lodz.pl

**Keywords:** melanoidins, *theobroma cacao* L., total phenolic compounds, antioxidant capacity, metal-chelating ability, fourier transform infrared spectroscopy

## Abstract

Melanoidins from real foods and model systems have received considerable interest due to potential health benefits. However, due to the complexity of these compounds, to date, the exact structure of melanoidins and mechanism involved in their biological activity has not been fully elucidated. Thus, the aim of this study was to investigate the total phenolic content, antioxidant properties, and structural characteristics of high-molecular weight (HMW) melanoidin fractions isolated by dialysis (>12.4 kDa) from raw and roasted cocoa beans of Criollo, Forastero, and Trinitario beans cultivated in various area. In vitro antioxidant properties of all studied HMW cocoa fractions were evaluated by four different assays, namely free radical scavenging activity against DPPH^•^ and ABTS^•+^ radicals, ferric reducing antioxidant power (FRAP), and metal-chelating ability. Additionally, the structure–activity relationship of isolated HMW melanoidin fractions were analyzed using attenuated total reflectance Fourier transform infrared spectroscopy (ATR-FTIR). The results show that roasting at a temperature of 150 °C and a relative air humidity of 0.3% effectively enhances the total phenolics content and the antioxidant potential of almost all HMW cocoa melanoidin fractions. The ATR-FTIR analysis revealed that the various mechanisms of action of HMW melanoidins isolates of different types of cocoa beans related to their structural diversity. Consequently, the results clearly demonstrated that HMW cocoa fractions isolated from cocoa beans (especially those of Criollo variety) roasted at higher temperatures with the lower relative humidity of air possess high antioxidant properties in vitro.

## 1. Introduction

In recent years, a number of biological properties and potential health benefits of consuming cocoa-derived products have been investigated, e.g., antioxidant, anti-inflammatory, anti-carcinogenic, and antifungal properties. Much research has focused on the cocoa phenolic compounds, such as flavonoids (procyanidins, anthocyanins, flavonols, etc.), as a potential health-promoting compounds due to its antioxidant capacity and abundance in the cocoa beans. However, the bioactive compounds composition of cocoa beans is influenced by many factors including variety, climatic and agronomic conditions, post-harvest practices, and storage conditions [1,2,3]. Moreover, thermal processing may also cause a change in the level of phytochemicals in cocoa beans and their antioxidant capacity. Many studies have shown that phenolic compounds, which have been suspected as primarily factors responsible for the antioxidant properties of cocoa beans, are susceptible to degradation and oxidative condensation during thermal processing [2,4,5,6,7]. A recent study showed that, beside loss of phenolic compounds, roasting of cocoa beans induced only negligible changes in the total antioxidant capacity, probably due to the higher extractability of the cellular matrix compounds and/or formation of new antioxidants trough Maillard reactions, such as reductones and melanoidins [5,6,8]. Melanoidins are colored, nitrogen-containing, and polymeric compounds that form as a result of the final stage of the Maillard reactions. These compounds occur widely in many treated processed foods, such as coffee, bakery products, cooked potatoes, cocoa, roasted barley, and beef [9]. One of the most important properties that melanoidins contribute to foodstuffs is brown color. In addition, these macromolecules have considerable structural variability that result in diverse biological effects. The interest in the physiological role of melanoidins present in many heat-treated foods has increased dramatically over the last decade, particularly in relation to human health [2,9,10]. The biological effects exerted by melanoidins on the human body are thought to be strongly related to their ability the chelate metal cations, the capacity of scavenging superoxide anions and hydroxyl radicals, and the decomposing ability of hydrogen peroxide [10,11], which may be responsible for the antioxidant, anticancer, and antimicrobial properties [12]. Food melanoidins can also act as dietary fiber in the gastrointestinal tract and promote the growth of beneficial *Bifidobacteria* in the gut [9,11]. Current studies have shown that these biological functions are thought to be associated, at least in part, with the presence of phenolic compounds in the melanoidin structure [11,13]. Phenolic compounds, especially phenolic acids, are considered to contribute more to the healthful effects than the other constituents of melanoidins [13,14]. However, the exact mechanism of melanoidins antioxidant activity has not fully been elucidated to date. Moreover, despite numerous studies evaluating the biological and molecular properties of melanoidins obtained from both model systems and real foodstuffs, such as coffee, bread, honey, heated potato fiber, and malt [9,11,15], only very few authors have attempted to determine the biological activities of melanoidins isolated from cocoa beans and cocoa-derived products. Summa et al. [16,17] investigated the antibacterial, mutagenic, and radical-scavenging effects of four molecular weight fractions (>30, 30–10, 10–5, and <5 kDa) isolated by ultrafiltration from raw, pre-roasted (80–90 °C for 10 min), and roasted (130–160 °C for 15–20 min) cocoa beans. In those experiments, all high-molecular weight (HMW) fractions showed activity against the pathogenic bacteria *Enterobacter* and *Escherichia*. More recent studies have reported that LMW (<10 kDa), intermediate molecular weight (10–30 kDa), and HMW (>30 kDa) fractions isolated from cocoa powder by ultrafiltration have a dose-dependent inhibitory activity against α-glucosidase [18]. This activity was attributed to the presence of compounds in the low, intermediate, and high molecular weight fractions, including brown melanoidins, proteins, phenolic compounds, as well as polysaccharides, whether or not they were bound to the melanoidin skeleton or to unknown MRPs [18]. In our previous study, we investigated the effect of different roasting conditions, including temperature (110, 120, 135, or 150 °C) and relative air humidity (0.3% or 5.0%) on the physicochemical properties and the profiles of free phenolics and bound phenolics of high molecular weight Maillard reaction products isolated from Criollo, Forastero, and Trinitario beans from different regions of Africa [19,20]. In our previous work, we also demonstrated that the of the cocoa melanoidins are good sources of bound phenolics [19]. In that respect, it is noteworthy that the relation between the structure of these compounds and their health-related properties should be comprehensively investigated. Especially when, for some groups of people, cocoa beans represent a significant source of these compounds in their diet, the accurate assessment of the contribution of the chemical and structural properties of melanoidins to their antioxidant activity should be carried out.

In continuation with our previous study, this work was designed to carry out a comparative investigation of total phenolic content, antioxidant activity, and structural characteristic of HMW melanoidin fractions isolated from cocoa beans, both raw and roasted at different temperatures (110, 120, 135, and 150 °C) and relative air humidity levels (RH 0.3% and 5.0%) of three *Theobroma cacao* L. types. To the best of our knowledge, this is the first report concerning the determination of in vitro antioxidant activity and ATR-FTIR structural characterization of isolated HMW melanoidin fractions of Criollo, Forastero, and Trinitario beans from different regions of Africa.

## 2. Materials and Methods

### 2.1. Materials and Chemicals

Gallic acid, 6-hydroxy-2,5,7,8-tetramethylchroman-2-carboxylic acid (Trolox), 2,2′-azino-bis (3-ethylbenzothiazoline-6-sulfonic acid) diammonium salt (ABTS), 2,2-diphenyl-1-(2,4,6-trinitrophenyl) hydrazyl (DPPH), 2,4,6-tri(2-pyridyl)-s-triazine (TPTZ), sodium acetate, ferric chloride hexahydrate, ferrozine, and ammonium acetate were purchased from Sigma-Aldrich (St. Louis, MO, USA). All other reagents were of analytical grade and were purchased from Chempur (Piekary Slaskie, Poland). Ultrapure water (resistivity 18.2 MΩ cm), obtained from a Milli-Q purification system (Millipore, Bedford, MA, USA), was used for all analyses.

### 2.2. Plant Material

Analyses and experiments were conducted on samples of fermented and dried cocoa beans of the three main *T. cacao* groups: Criollo and Trinitario originating from Madagascar, and Forastero from Ghana. All cocoa beans were harvested at their technological maturity in 2013 and purchased from commercial sources. Cocoa fruit reaches physiological maturity around 5 months after flowering and is harvested at 5–6 months. Cocoa pods harvested at 5–6 months are considered to be technologically matured as the beans have developed optimum quality [21]. The description of cocoa beans is presented in Table 1.

Raw cocoa beans of each group after removal of impurities and broken or chipped beans were convectively roasted in batches of 200 g in a tunnel with the forced air flow without circulation (adapted for either dry or humid air). Process air humidity was gradually increased using 4.0 MPa saturated steam from a generator. Roasting was performed at four temperatures (110, 120, 135, or 150 °C) and two relative air humidity levels (0.3% or 5.0%). The heat treatment parameters were chosen to obtain a range of roasted beans with acceptable physico-chemical and sensory properties. Usually, during roasting, the raw cocoa beans are exposed to temperatures that range from 135 to 150 °C, whereas the “fine or flavor” varieties require lower temperatures than the “bulk” ones [19]. It was terminated when bean moisture dropped to 2% as determined by drying until constant weight at 103 ± 2 °C. The time of thermal treatment was determined experimentally for each batch of cocoa beans, based on their initial water content and size. Roasting times at 150, 135, 120, and 110 °C were approximately 20, 40, 75, and 85 min, respectively. The application of higher relative air humidity prolonged the duration of thermal treatment. At the end of roasting, the beans were immediately cooled to approximately 20 °C for about 10 min. The roasted cocoa beans were kept in hermetically sealed plastic containers (500 g) and stored at −20 °C for subsequent analyses. The samples were analyzed within 6 to 12 h of storage. All roasting experiments were performed in duplicate for each cocoa type used.

### 2.3. Extraction and Isolation of Cocoa Melanoidin

The HMW melanoidin fraction was obtained from raw and roasted cocoa beans by dialysis according to our previously research [19]. Briefly, the cocoa beans were deshelled, ground, and defatted, and then extracted twice with 100 mL of water at 90 °C for 20 min in an orbital shaker (100× *g*). Subsequently, pooled extracts were cooled to room temperature and filtered through Whatman no. 4 filter paper to remove insolubles. An aliquot of the filtrate was dialyzed using a dialysis tubes (MW cut-off > 12.4 kDa, Sigma-Aldrich, Saint Louis, MO, USA) for 1 day under running tap water and for 2 days against 2000 mL of water at 4 °C with constant stirring. The water was changed 4–5 times until no further color was visible in the dialysate. The dialysate containing LMW compounds was removed. After dialysis, the retentate containing the HMW fraction was frozen at −20 °C and lyophilized (−50 °C, 0.9 MPa) using a DELTA 1-24LSC Christ freeze drier (Martin Christ, Osterode am Harz, Germany). All lyophilized HMW materials were then stored in plastic bags at −20 °C to prevent hydration until used.

### 2.4. Determination of Total Phenolic Content

Total phenolic contents (TPC) of HMW isolates from raw and roasted cocoa beans were determined using the Folin–Ciocalteu method, as described by Belšcak et al. [22]. Briefly, 100 µL of the suitably diluted lyophilized HMW cocoa sample with high-purity deionized water (1.5 mg/mL) or blank was mixed with 4 mL of high-purity deionized water and 0.5 mL of the Folin–Ciocalteu reagent. After 3 min, 1 mL of 20% (w/v) Na_2_CO_3_ solution was added to the mixture. The final volume was adjusted to 10 mL with high-purity deionized water. The solution was then mixed vigorously and allowed to stand at room temperature in the dark place for 60 min. The absorbance of reaction mixture was measured at 765 nm using a UV-1800 spectrophotometer (Shimadzu, Tokyo, Japan). For each sample, experiments were conducted in triplicate. The results were expressed as mg gallic acid equivalents (GAE) per gram of lyophilized HMW cocoa fraction dry weight (mg GAE/g dw).

### 2.5. Determination of the Free Radical-Scavenging Capacity

The free radical-scavenging activity of the HMW isolates from raw and roasted cocoa beans was determined by the DPPH and ABTS assays as previously described by Oracz and Nebesny [8]. All analyses were carried out in triplicate, and the results obtained from the two tests were expressed as µmol Trolox equivalents per gram of lyophilized HMW cocoa fraction dry weight (μmol TE/g DW).

### 2.6. Determination of Ferric Reducing Antioxidant Power (FRAP)

The FRAP assay was performed according to the protocol described by Pastoriza and Rufián-Henares [23] with slight modification. The fresh working FRAP solution was prepared by mixing 25 mL of 300 mmol/L acetate buffer (pH 3.6), 2.5 mL of 10 mmol/L TPTZ in 40 mmol/L HCl, and 2.5 mL of 20 mmol/L FeCl_3_·6H_2_O and then warmed at 37 °C for 30 min before using. Aliquots (0.1 mL) of the suitably diluted lyophilized HMW cocoa samples with high-purity deionized water (1.5 mg/mL) were mixed with 4 mL of fresh FRAP solution. The solution was then mixed vigorously and allowed to stand for 30 min at 37 °C. The absorbance of reaction mixture was measured at 593 nm using a UV-1800 spectrophotometer (Shimadzu, Tokyo, Japan). For each sample, experiments were conducted in triplicate. The results were expressed as µmol Trolox equivalents per gram of lyophilized HMW cocoa fraction dry weight (μmol TE/g dw).

### 2.7. Determination of Chelating Activity on Fe^2+^

Chelating activity of the HMW isolates from raw and roasted cocoa beans was evaluated using the method of Gu et al. [24]. Briefly, 1 mL of the suitably diluted lyophilized HMW cocoa materials in high-purity deionized water (2 mg/mL) were added to 1.85 mL of high-purity deionized water and 50 μL of 2.0 mM FeCl_2_. After mixing, the solution was allowed to stand at room temperature for 30 s, followed by the addition of 100 μL 5 mM ferrozine. The reaction mixture was then vortexed and left to stand at room temperature for 15 min. The absorbance of the solution was measured spectrophotometrically at 562 nm with UV-1800 UV-VIS Spectrophotometer (Shimadzu, Tokyo, Japan). Worth noting, that low absorbance of the resulting solution indicated a strong ferrous ion chelating ability. A reaction mixture containing 1 mL of high-purity deionized water instead of sample solution served as the blank. The results are expressed as the percentage chelating activity (%).

### 2.8. Attenuated Total Reflection Fourier Transform Infrared Spectroscopy

The infrared spectroscopy is based on the absorption of radiation due to vibrations bonds of molecules [25,26]. The FTIR spectra of lyophilized HMW cocoa melanoidin fractions were obtained using an infrared Fourier transform spectrometer, model IRTracer-100 (Shimadzu, Tokyo, Japan) equipped with an attenuated total reflection (GladiATR) accessory with diamond crystal (PIKE Technologies, Inc., Madison, Wisconsin, USA) at room temperature. The spectral range was 400–4000 cm^−1^ with 40 scans and a resolution of 4 cm^−1^. Around 5 mg of lyophilized HMW cocoa fractions was deposited on the diamond platform prior to measurement. Background and sample spectra were acquired at 4 cm^−1^ resolution with 40 scans from 400 to 4000 cm^−1^

### 2.9. Statistical Analysis

The results are presented as mean ± standard deviations of three replicates. Statistical tests were evaluated by using the Statistica 13.0 software (StatSoft, Inc., Tulsa, OK, USA). The obtained data were tested for normal distribution (Shapiro-Wilk test) and equal variances (Levene’s test). As all values showed normal distribution and homogeneity of variance, considering *p* ≥ 0.05, the data were subjected to the analysis of variance (ANOVA). The effects of variety, roasting temperature, roasting air humidity, and their interaction on total phenolic content and antioxidant activity of HMW melanoidin fractions were tested by means of two-way ANOVA. The significant differences among the means were estimated through Tukey’s HSD test. For all statistical analysis, *p* < 0.05 was considered as statistical significance. The error bars in all figures correspond to the standard deviations. The correlation coefficients between investigated parameters were assessed by means of the Pearson correlation test using Microsoft Office Excel 2016 (Microsoft Corporation, Redmond, WA, USA).

## 3. Results and Discussion

The present study is a continuation of our previous work [19,20] exploring the physicochemical properties and the profiles of free and bound phenolics of HMW melanoidin fractions of different *T. cacao* groups and origins. In this work, the total phenolic content and the antioxidant activity of the HMW melanoidin fractions (>12 kDa) from raw and roasted, at different temperatures and relative air humidities, cocoa beans of different groups were evaluated by using different in vitro spectrophotometric assays.

### 3.1. Total Phenolics Content

**Figure 1:**
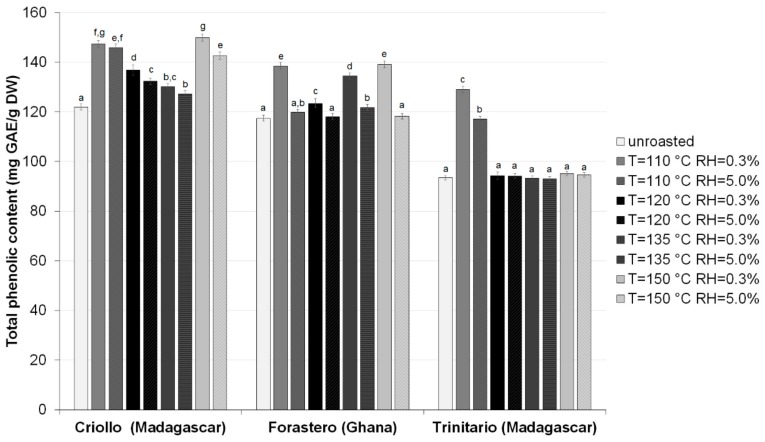
Total phenolics content of melanoidin fractions isolated from raw and roasted, at different at different temperatures and relative air humidities, cocoa beans of different groups. Results are presented as means ± SD from triplicate assays. Bars with the same lowercase letter (a–g) within each variety do not differ significantly according to Tukey’s HSD test at *p* < 0.05.

The total phenolics content (TPC) of HMW melanoidin fractions from the three cocoa types is shown in Figure 1. The data were expressed as milligrams of gallic acid equivalents (GAE) per gram dry weight (mg GAE/g dw). In this study, significant differences (*p* < 0.05) in TPC of the HMW fractions were found between the three cocoa types. The HMW fractions from both raw and roasted Criollo beans were characterized by the highest TPC, ranging from 121.99 to 149.90 mg GAE/g DW. Less phenolics were observed in HMW isolates from Forastero beans (117.46–139.12 mg GAE/g DW), while HMW materials from Trinitario beans contained the lowest amounts of these compounds (93.02–129.00 mg GAE/g DW). In order to assess the influence of cocoa variety, roasting temperature, roasting air humidity, and their interactions on TPC content of HMW melanoidin fractions, a two-way ANOVA was carried out. The results showed that the cocoa variety and roasting process parameters significantly affected the TPC of HMW melanoidins isolated from the studied cocoa beans (*p* < 0.01). Generally, more phenolics were contained in the almost all of HMW fractions isolated from roasted beans than from the raw ones. The greatest increase in TPC (by 25.3–38.1% of initial value) of the HMW fractions was observed when cocoa beans of the Trinitario type were roasted at 110 °C. Moreover, it was also noticed that thermal processing of Criollo and Forastero beans at 150 °C with RH of 0.3% led to the highest increase in the TPC (by 18.4–22.9% of initial value). As can be seen from the results, the changes in the TPC of the HMW fractions also depended on the RH and in almost all samples were slightly less advanced when the air humidity was increased from 0.3% to 5.0%. Thus, it was confirmed that influence of the roasting parameters on TPC of the HMW fractions is complex, and huge differences are observed depending on applied cocoa bean variety and process conditions, like temperature and relative air humidity level. According to Pérez-Martínez et al. [27], the Folin–Ciocalteau assay measures the reducing capacity of a sample, and the results of TPC may be affected by the presence of other electron donors, such as non-phenolic substances and nitrogen-containing compounds. Thus, the increase in the level of compounds able to react with the Folin–Ciocalteu reagent could be linked to the formation of new substances, especially reductones, during the roasting of cocoa beans. These compounds, which are formed in the advanced and final stages of Maillard reaction, can act as reducing agents, due to the presence of hydroxyl and pyrrole groups [24,28].

Summa et al. [16] reported that pre-roasting of cocoa beans (at 80–90 °C for 10 min) significantly reduced the concentration of Folin–Ciocalteu reactive substances in the 30–10 kDa fraction while accurate roasting (at 130–160 °C for 15–20 min) increased their content. As suggested by these authors, this phenomenon might be caused by formation of reducing substances in the 30–10 kDa fraction during roasting at high temperatures. They also found that the level of reducing compounds in the >30 kDa fraction increased significantly during cocoa beans pre-roasting but declined markedly after accurate roasting. In addition, the observed behavior can be a consequence of the incorporation of phenolic compounds into the structure of melanoidins during heat treatment [15,29]. The oxidized polyphenols may react with thiol groups on amino acids, peptides, and proteins or nucleophilic amines via Michael-type addition or Schiff base reaction to form thiol−quinone and amine–quinone adducts or benzoquinone imines [15,30,31,32]. Our previous studies have shown that roasting of cocoa beans of different types at temperatures ranging from 110 to 150 °C led to a marked increase in the bound phenolics content as compared to HMW fractions from unroasted beans [20]. Therefore, we can concluded that significant differences in the TPC observed between the HMW cocoa melanoidin fractions appeared to be brought about by the structural changes of their active components, involving oxidation of phenolic compounds, their condensation with proteins and polysaccharides, as well as cross-linking and polymerization of low molecular weight MRPs during roasting, which decide their electron donating abilities [20,31,33].

### 3.2. Free Radicals-Scavenging Capacity

To make a comprehensive evaluation on the antioxidant effect of samples, it is necessary to employ different methods due to the fact that this assays exhibit different mechanisms of by which its antioxidant activity takes place. In vitro free radical scavenging activities of the HMW melanoidin fractions from raw and roasted at various conditions cocoa beans of different *T. cacao* groups and origins were assessed against DPPH and ABTS radicals (Table 2).

The ABTS•^+^ and DPPH assays are widely used methods for the assessment of the antioxidant activities of many vegetable or food matrices [34,35,36,37]. These methods are both based on quenching of stable colored radicals and show the free radical quenching activity of antioxidants even when present in complex biological matrices such as plant or food preparations (extracts or fractions). The values of antioxidant potential obtained in both assays were equal or higher than those reported by other authors for the HMW melanoidin fractions of cocoa beans, chocolate, or even coffee [16,23,38], meaning that all of studied HMW cocoa melanoidin samples exhibited good DPPH and ABTS radical scavenging activities.

However, there were significant differences between HMW melanoidin fractions from both the raw and roasted cocoa beans regarding their antioxidant abilities. Two-way ANOVA revealed significant effect of cocoa variety (*p* < 0.01), roasting temperature (*p* < 0.001), roasting air humidity (*p* < 0.01), and interaction of variety and roasting conditions (*p* < 0.001) for the DPPH radical-scavenging activity (Table 2). In addition, ABTS scavenging capacity were significantly affected by variety, roasting temperature, and interaction of variety and roasting conditions, but the levels of relative humidity did not have significant influence. The HMW materials from unroasted Criollo beans demonstrated the highest scavenging capacity against both DPPH and ABTS radicals (726.26 and 849.84 μmol TE/g dw, respectively). Compared with HMW melanoidins from Criollo beans, the HMW fraction from unroasted Forastero beans showed the significantly lower (*p* < 0.05) activity against DPPH (689.16 μmol TE/g dw) but the similar against ABTS radical cations (806.22 μmol TE/g dw). The lowest radical scavenging abilities against the ABTS and DPPH assays was exhibited by HMW isolates from unroasted Trinitario beans, which is consistent with the results of TPCs discussed above. These results showed that, depending on the cocoa type and roasting conditions, an increase or decrease in the antioxidant capacity of HMW melanoidin fractions was observed. Roasting of all types of cocoa beans at temperatures ranging from 110 to 150 °C caused a significant increase (*p* < 0.05) in the DPPH radical-scavenging activity of HMW fractions. However, the changes in the free radical-scavenging activity of HMW fractions were considerably smaller at the higher RH. Interestingly, we observed that HMW fractions from Criollo beans roasted at 150 °C with lower RH exhibited a higher antioxidant activity by DPPH scavenging assay (1475.61 μmol TE/g dw) compared to all other melanoidin samples. Roasting of Forastero and Trinitario beans at temperatures ranging from 110 to 150 °C caused a significant increase (*p* < 0.05) in the ABTS radical-scavenging activity of their HMW melanoidin fractions. It was also observed that the HMW fractions from Criollo beans, roasted at temperatures of 110, 120, and 150 °C, displayed the significantly increased ABTS scavenging capacity, while after roasting at 135 °C, exhibited a slightly lower antioxidant activity by this method. Nevertheless, among HMW melanoidin fractions isolated from roasted beans of the three cocoa varieties, the highest ABTS radical-scavenging capacity (1172.37 μmol TE/g dw) was exhibited by the fraction from Criollo beans roasted at 120 °C and RH of 0.3%. Based on the results presented in Table 2, it is possible to observe that, in almost all HMW fractions, antioxidant activity is higher in the DPPH method (except HMW fractions of Criollo beans roasted at 110 °C). This behavior is similar to the observed by other researchers [23] who have observed a stronger scavenging activity against DPPH^•^ than ABTS•^+^ radicals in the case of chocolate melanoidins. Our results corroborated also with those reported by Summa et al. [16], who showed that the >30 kDa fraction obtained from pre-roasted cocoa beans had higher DPPH and ABTS radical-scavenging activity compared to the >30 kDa fractions of raw and roasted beans. The differences between the ABTS and DPPH radical-scavenging activity could be explained by the wide variety of chemical mechanisms involved in the antioxidant activity of HMW cocoa melanoidin fractions, as it was mentioned earlier. The reaction mechanism with DPPH^•^ radical involve transfer of a hydrogen atom, while the reactions with ABTS•^+^ radicals involve electron transfer process [39]. The observed differences indicate the complexity of the mechanism of action of melanoidins formed in real food. The increase in the antioxidant capacity of the HMW fractions can be a consequence of accumulation of high molecular weight MRPs-like melanoidins during roasting. In addition, the observed behavior can be explained by the presence of residues of certain active compounds, containing more than one active group (OH or NH_2_), such as phenolic compounds, quinones, and low molecular weight MRPs, in the HMW materials. These compounds might be attached to the structure of melanoidins via non-covalent bonds and influence their biological properties [4,5,18]. Although the content of free phenolic compounds in HMW fractions of roasted cocoa beans decreased [19], it is possible that it could be explained by the presence of quinones generated by oxidation of these compounds that spontaneously form covalent bonds to functional groups of melanoidins during roasting [4,40]. We suppose that the radical-scavenging activity of tested HMW fractions from cocoa beans depends on the structure and the number of the included active group (OH or NH_2_). This was a clear indication that the synergistic effect between different bioactive compounds present in the structure of melanoidins could determine their biological properties. Since, the HMW cocoa melanoidins have the ability to scavenge free radicals, thereby preventing lipid peroxidation chain reactions that cause damage a wide range of molecules found in living cells, they could serve as potential nutraceuticals and functional ingredients.

### 3.3. Ferric Reducing Antioxidant Power

The reducing capacity may serve as a significant indicator of potential antioxidant activity. Thus, the HMW melanoidin fractions of unroasted and roasted cocoa beans of tree different *T. cacao* beans was estimated for their ability to reduce TPTZ–Fe (III) complex to TPTZ–Fe (II). In order to identify the influence of cocoa variety, roasting temperature, roasting air humidity, and their interactions on ferric reducing antioxidant power (FRAP), a two-way ANOVA was performed. As demonstrated in Table 2, significant differences (*p* < 0.05) in the reducing properties of HMW fractions were found between the three cocoa types. The HMW fractions from Criollo beans exhibited the highest FRAP values, ranging from 622.42 to 1077.18 μmol TE/g dw, followed by isolates of Forastero beans (533.90–947.46 μmol TE/g dw), while HMW materials of Trinitario beans had the lowest reducing power (440.49–779.49 μmol TE/g dw). The results also showed that the roasting process parameters significantly (*p* < 0.05) affected the reducing properties of HMW materials isolated from the studied cocoa beans. Generally, all of HMW fractions isolated from unroasted beans showed a lesser reducing power compared to the isolates from the roasted samples. The greatest increase in the ferric reducing ability (by 71.1%–77.0% of initial value) of HMW fractions was observed when cocoa beans of all studied cocoa groups were roasted at 135 °C with RH of 0.3%. As can be seen from the results, the changes in the reducing power of HMW fractions depended on the RH and in almost all samples was slightly more pronounced when the air humidity was decreased from 5.0% to 0.3%. We can conclude that all studied HMW cocoa melanoidin fractions behaved as reductants and inactivators of oxidants due to the presence of electron-donors in their structure. The observed increase in the reducing capacity of HMW cocoa fractions could be linked to the formation of new substances able to donate electrons or to terminate radical chain reactions, during roasting of cocoa beans. As described above, the HMW cocoa melanoidin fractions contained different amounts of free and bound phenolic compounds and the presence of these polyphenols along with compounds formed in the advanced and final stages of Maillard reaction could contribute to the antioxidant properties observed by FRAP method. These residues due to the presence of hydroxyl and pyrrole groups can act as reducing agents via their redox potential of transferring electrons [24,33]. In a study performed by Summa et al. [16], it was observed that roasting of cocoa beans (at 130–160 °C for 15–20 min) significantly increased the concentration of reducing substances in the 30–10 kDa fraction. They also found that the level of reducing compounds in the >30 kDa fraction increased significantly during cocoa beans pre-roasting but declined markedly after roasting.

### 3.4. The Weighted Average Antioxidant Capacity

**Figure 2:**
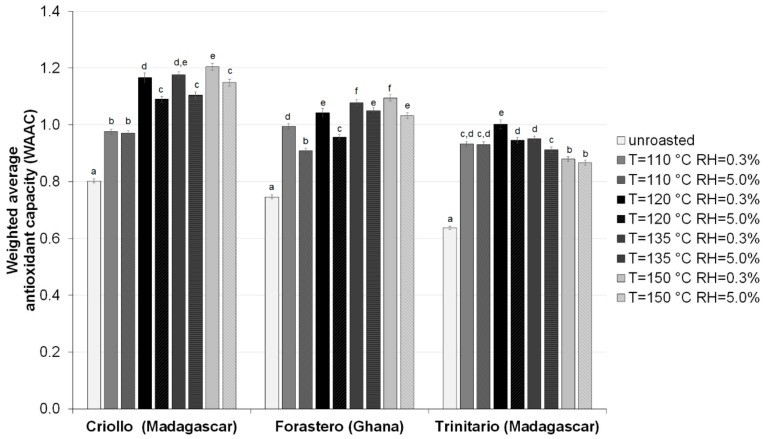
The weighted average antioxidant capacity of HMW fractions isolated from raw and roasted, at different at different temperatures and relative air humidities, cocoa beans of different groups. Results are presented as means ± SD from triplicate assays. Bars with the same lowercase letter (a–f) within each variety do not differ significantly according to Tukey’s HSD test at *p* < 0.05.

Our results suggest that the antioxidant capacity of HMW fractions from raw and roasted beans depend on the cocoa type and roasting conditions, wherein no significant correlation was observed between DPPH, ABTS, and FRAP values. This may be explained by the fact that different constituents in the HMW fractions would have different mechanisms of action regarding their antioxidant activities. According Tabart et al. [41], the weighted average of the results obtained by the different assays should be calculated to get an overall impression of the antioxidant potential of the samples. Therefore, the results of antioxidant capacity of HMW fraction obtained by the specific assay (DPPH, ABTS, and FRAP) was divided by the average activity of the all HMW samples by the same assay, and then the calculated values in each assay were summarized and divided by the number of assays used (three in our case). The weighted average antioxidant capacity of each HMW samples are shown in Figure 2. The results of the present study show that cocoa beans roasting temperatures of 110–150 °C cause significant rise in the weighted average antioxidant capacity (WAAC) of the HMW materials. The highest WAAC values were obtained at a roasting temperature of 150 °C and RH of 0.3% (in the case of Criollo and Forastero groups) and a roasting temperature of 120 °C and RH of 0.3% (in the case of Trinitario group). It was also observed that the HMW fractions of both raw and roasted Criollo beans exhibited the highest WAAC values. The HMW fractions derived from almost all beans of Forastero type has a slightly higher WAAC compared to the samples of Trinitario type (except for beans roasted at 110 °C and RH of 5.0%). This is reflected in the results of the two-way ANOVA (Figure 2) indicating that WAAC was significantly affected by the cocoa variety (*p* < 0.001), roasting temperature (*p* < 0.001), roasting air humidity (*p* < 0.01), and their interactions (*p* < 0.001).

### 3.5. Chelating Activity on Fe^2+^

**Figure 3:**
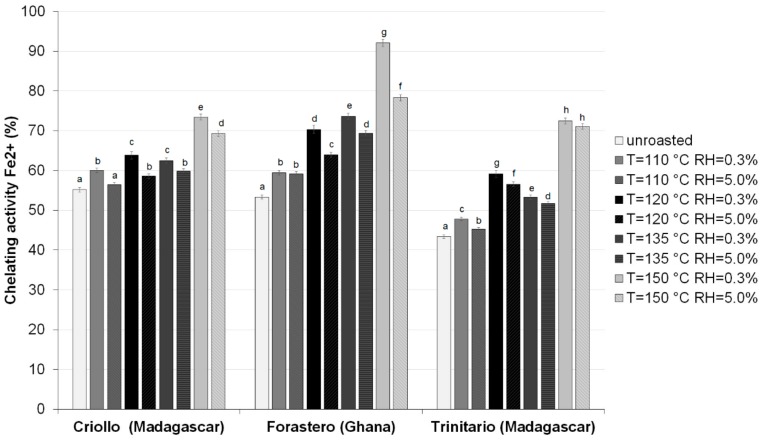
Total phenolics content of HMW fractions isolated from raw and roasted, at different at different temperatures and relative air humidities, cocoa beans of different groups. Results are presented as means ± SD from triplicate assays. Bars with the same lowercase letter (a–h) within each variety do not differ significantly according to Tukey’s HSD test at *p* < 0.05.

The changes in ferrous ion chelating activity of HMW fractions, caused by roasting of beans of all tested cocoa groups, are presented in Figure 3. All HMW isolates obtained from raw and roasted cocoa beans were able to chelate ferrous ion (Fe^2+^), being the most powerful pro-oxidants among various species of metal ions. The ferrous ion in the Fenton reaction can catalyze the generation of potentially toxic reactive oxygen species (ROS), such as hydroxyl radicals (•OH) that initiate lipid peroxidation [28]. It was also found that, similar to the WAAC, the HMW fractions from raw Criollo beans showed the higher chelating activity (55.16%) than those derived from raw Forastero and Trinitario beans (53.31% and 43.39%, respectively). To the best of our knowledge, this is the first study showing the interplay between roasting conditions and the iron chelating activity of HMW fractions isolated from different groups of cocoa beans. Two-way ANOVA revealed that the effect of cocoa variety, roasting conditions, and their interactions was highly significant (*p* < 0.001). The results of this study demonstrate that the chelating activity of HMW fractions isolated from beans of the three cocoa types was increased by roasting at temperatures in the range of 110 to 150 °C, which may be ascribed to structural changes in melanoidins and phenolic compounds. Moreover, the changes in the iron binding ability greatly depended on the roasting air humidity (0.3–5.0%) and were considerably smaller when the RH was elevated. The chelating activity of the HMW isolates obtained from roasted samples varied from 45.21% to 92.07%. The increase in the metal-chelating activity was more pronounced in HMW isolates from Forastero and Trinitario beans, while the lowest changes were observed in the Criollo samples. Irrespective of the cocoa type, the greatest increase in the iron chelating ability occurred when cocoa beans were roasted at 150 °C and the lower relative humidity of air (RH of 0.3%) whereas the lowest rise was caused by roasting at 110 °C with humid air (RH of 5.0%). These results are consistent with the described above differences in the free radical scavenging activities and the reducing properties of HMW cocoa. However, unlike to the WAAC, the melanoidin fraction of Forastero beans roasted at higher temperatures exhibited the highest ferrous ion chelating potential, compared to other cocoa groups (even in the relation to Criollo samples).

Such differences could be explained by variations in the composition of studied HMW melanoidin fractions that are strongly determined by cocoa variety and roasting conditions [19,20]. In addition, this phenomenon suggests that Maillard reactions that took place during roasting of cocoa beans at the higher temperatures resulted in formation of high molecular weight brown melanoidins, exhibiting the high iron chelating activity. Our findings confirm recently reported observation that MRPs with metal-chelating ability are generated in model systems due to heat treatment. Gu et al. [24] found that high molecular weight MRPs had higher metal-chelating potential than low molecular weight MRPs. They also suggest that the metal-chelating activity of MRPs is possibly affected by the presence of the hydroxyl or pyrrole groups in their structures. This was a clear evidence that the studied HMW cocoa melanoidins may prevent against oxidative damage by sequestering Fe (II) ions that participate in transition metal ions-catalyzed hydrogen peroxide decomposition with generation of hydroxyl radicals.

### 3.6. Characterization of Cocoa Melanoidins by Fourier Transform Infrared Spectroscopy

**Figure 4:**
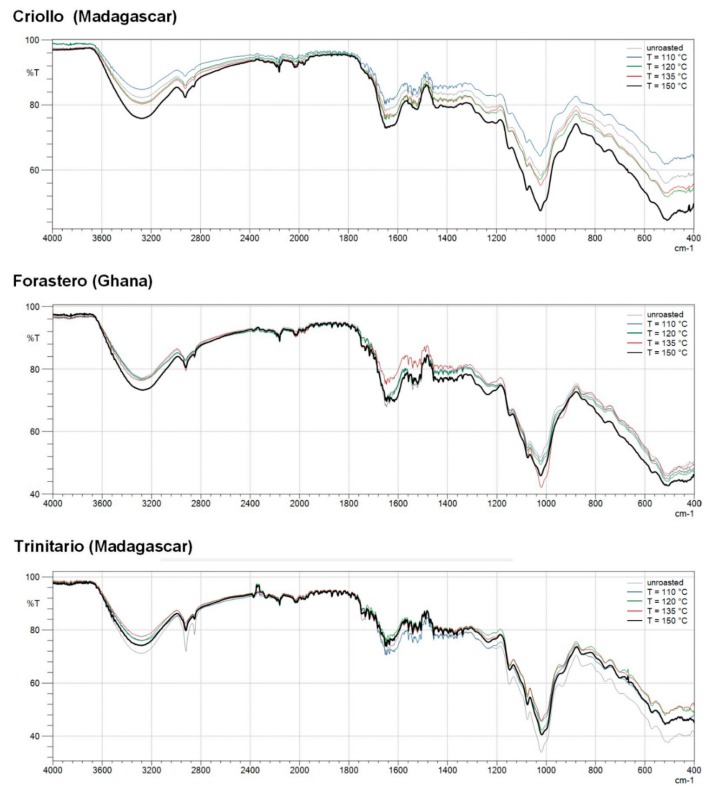
Fourier transform infrared spectroscopy (FTIR) spectra of HMW fractions isolated from raw and roasted, at different at different temperatures and relative air humidities, cocoa beans of different groups.

To provide a comprehensive explanation about the antioxidant properties of HMW cocoa fractions, it was necessary to precisely characterize the chemical structure of these materials. We report here for the first time the ATR-FTIR spectroscopic characterization of HMW cocoa melanoidins. The FTIR spectra of HMW isolates obtained from raw and roasted cocoa beans were collected with FTIR spectrometer equipped with an ATR sample accessory. The ATR-FTIR spectra (within the 400 cm^−1^ to 4000 cm^−1^ wavenumber region) of the cocoa melanoidins are given in Figure 4.

The heterogenic nature of the cocoa melanoidins has been confirmed by Fourier transform infrared spectra of HMW fractions isolated from different types of *T. cacao* beans, which exhibit prominent absorption bands in the broad region 400–4000 cm^−1^, the characteristic for various classes of compounds. Common features as well as particular vibrations, specific to from phenolic compounds and its derivatives, polysaccharides, and proteins, are found in the spectra. The ATR-FTIR spectra of all HMW cocoa melanoidin fractions contains a wide band at 3350 cm^−1^ that belongs to the H-bond stretching vibrations of O–H hydroxyl groups, strong stretching band at about 1650 cm^−1^ assigned to the double bond stretching of carbonyl (C=O), C=C, or C=N [42] and characteristic absorption bands appeared between 900–600 cm^−1^ due to the stretching vibrations of the entire anhydroglucose ring. All mentioned bands confirms the presence of a phenol group sensitive to hydrogen bonding [25]. Moving forward, it confirms that more phenolic compounds bound to melanoidins might also contribute to the observed strong antioxidant capability of HMW cocoa fractions. The antioxidant activity of phenolic compounds is attributed to its molecular structures, particularly the number and positions of the hydroxyl groups, and the nature of substitutions on the aromatic rings [43]. Therefpre, more active antioxidant compound possesses more hydroxyl groups. The changes in the spectra gradually occur, depending on the cocoa type and roasting conditions. Our results showed that thermal treatment temperatures between 110 and 150 °C generally caused an increase in intense of the peak of HMW fractions isolated from cocoa beans (except for Trinitario samples). The peaks at 2922 and 2850 cm^−1^ in the FTIR spectra were attributed to the stretch of the C–H of aromatic ring and would be due to stretching vibrations of CH_2_ and CH_3_ groups that can originate from fatty acids present in the cocoa melanoidin fractions [25]. A igher peak in the region 2920 cm^−1^ clearly reflects adsorption of aliphatic compounds in the melanoidins structure. Some reports revelated that interactions between reactive carbonyl compounds arising from lipid oxidation reactions and amino acids or proteins might play an important role in the formation of brown HMW macromolecular compounds upon high-temperature processing of cocoa beans [44,45]. Bands in the range of 1616–1690 cm^−1^ were ascribed to N–H bending vibrations from amine or amide groups, and C=O stretching vibrations from flavonoids, phenolic acids and its derivatives, quinones, and lipids [45,46]. The band centered at about 1650 cm^−1^ mainly corresponds to the C=O stretching vibrational mode of the different structures of the protein backbone. The band at 1515 cm^−1^ was attributed to C=C stretching vibrations from aromatic rings of phenolic compounds [25,26,45]. Mot [46] noticed that the intensity of the peak at 1630 cm^−1^ is closely correlated with the antioxidant activity of plant extracts. In this study, in the FTIR spectrum of HMW fractions of Trinitario beans, the intensity of the 1630 cm^−1^ peak is significantly lower compared to the Criollo and Forastero melanoidins. This behavior corroborates the results obtained in DPPH˙ and ABTS scavenging assays and could be a consequence of the higher number of phenolic groups in the HMW melanoidin fractions of Criollo and Forastero groups compared to those of Trinitario type. Moreover, the relative intensity of this peak increases during roasting of almost all cocoa beans, which may be the result of adsorption of phenolic compounds and/or its derivatives in the melanoidin structures. This was a clear evidence that the studied HMW melanoidin fractions possessed residues (OH or NH_2_ groups) that could act simultaneously as hydrogen and electron donors. Our previous study on UHPLC-DAD-ESI-HR-MS^n^ analysis of HMW melanoidin fractions derived from two different types of cocoa beans revealed that both free and bound phenolic compounds, including three flavan-3-ols, seven phenolic acids, one phenolic aldehyde, and four *n*-phenylpropenoyl-L-amino acids (NPAs), are present in these fraction [20]. Most of the phenolics in all the HMW melanoidin fractions were present in the bound form. It was also found that HMW fractions obtained from roasted cocoa beans had the higher content of bound phenolics than those from unroasted cocoa beans. This observation is consistent with the results of other authors who showed that phenolic compounds can be incorporated into the melanoidin skeleton during coffee roasting [15]. The spectral region from 1400 to 650 cm^−1^ is called the fingerprint region. This region of the infrared spectrum contains vibrations that are specific for the large number of infrared bands, including C–O, C–C, and C–N single bond stretches, C–H bending vibrations, and some bands due to benzene rings [25,26]. The peaks in the range of 1020–1060 cm^−1^ corresponded to C–O–C and C–O stretching vibrations of the glycoside linkage and C–O bond stretching vibration in glycerol. According to the literature, the occurrence of this band is an evidence of polysaccharide and lipids in samples [25,26].

Our results demonstrate that ATR-FTIR spectroscopy may be used as a direct and nondestructive method for the rapid investigation of the structural characteristics and functional properties of the HMW melanoidin fractions. According to our findings, the presence of different types of compounds in HMW melanoidin fractions is responsible for their bioactive properties (e.g., reducing power, antioxidant capacity, chelating activity) and its different mechanisms of action.

## 4. Conclusions

In conclusion, the present study, to the best of our knowledge, was the first time a comprehensive study was carried out on the total phenolic content and antioxidant activities, as well as structure–activity relationships of HMW cocoa melanoidin fractions isolated from raw and roasted, under different temperature and relative air humidity conditions, cocoa beans of different *T. cacao* groups. The results showed that the cocoa type and roasting conditions affect the total phenolic content and antioxidant properties of HMW melanoidin fraction isolated from the studied beans. The ATR-FTIR analysis revealed the presence of different bioactive compounds with various mechanism of action in HMW cocoa melanoidin fractions. We found that, both for TPC and in vitro antioxidant activity, HMW cocoa melanoidin fractions of Criollo beans showed significantly higher values than those of Fosrastero and Trinitario samples. Moreover, we observed that that roasting at higher temperatures with the lower relative humidity of air effectively enhances in vitro antioxidant potential of almost all HMW fractions isolated from cocoa beans. Consequently, irrespective of the cocoa type, the thermal processing at 150 °C and RH of 0.3% can be recommended to obtain HMW materials with the highest total phenolic content and strong in vitro antioxidant potential. This fact could indicate that the thermal processing of cocoa beans enhances the concentration of bioactive molecules in HMW fractions that contribute to the antioxidant response observed in in vitro tests. Our findings suggest, in general, that optimization of the roasting conditions and choosing an appropriate cocoa variety may provide functional advantages by enhancing in vitro antioxidant properties of HMW fractions of cocoa beans.

## Figures and Tables

**Table 1:** Selected physical characteristics of different cocoa varieties used in the study.

Variety	Country of Origin	Mean Size (Length × Width) (mm)	Mean Bean Weight (g)
Criollo	Madagascar	25.1 × 10.2	1.75 ± 0.07 ^c^
Forastero	Ghana	22.3 × 8.1	1.32 ± 0.12 ^a^
Trinitario	Madagascar	24.8 × 9.2	1.54 ± 0.08 ^b^

Means sharing the different letters (^a–c^) are significantly different according to Tukey’s HSD test at *p* < 0.05.

**Table 2:** The free radical scavenging capacity (2,2-diphenyl-1-(2,4,6-trinitrophenyl) hydrazyl (DPPH) and 2,2′-azino-bis (3-ethylbenzothiazoline-6-sulfonic acid) diammonium salt (ABTS)) and ferric reducing ability (FRAP) of high-molecular weight (HMW) fractions isolated from raw and roasted at different conditions cocoa beans of Criollo, Forastero, and Trinitario groups originating from various geographical regions.

Varieties (Country of Origin)	Roasting Conditions	DPPH	ABTS	FRAP
**Criollo** (Madagascar)	unroasted	726.26 ± 1.53 ^a^	849.84 ± 1.65 ^c^	622.42 ± 1.71 ^a^
	T = 110 °C, RH = 0.3%	902.61 ± 1.65 ^c^	920.74 ± 1.68 ^d,e^	846.75 ± 1.67 ^b,c^
	T = 110 °C, RH = 5.0%	826.77 ± 1.74 ^b^	943.17 ± 1.72 ^e^	869.94 ± 1.59 ^c^
	T = 120 °C, RH = 0.3%	1172.37 ± 1.76 ^e^	1127.53 ± 1.54 ^g^	915.42 ± 1.62 ^d^
	T = 120 °C, RH = 5.0%	1074.21 ± 1.71 ^d^	1086.72 ± 1.65 ^f^	841.03 ± 1.59 ^b^
	T = 135 °C, RH = 0.3%	1384.97 ± 1.68 ^g^	805.94 ± 1.59 ^b^	1077.18 ± 1.72 ^g^
	T = 135 °C, RH = 5.0%	1250.47 ± 1.72 ^f^	772.74 ± 1.73 ^a^	1036.51 ± 1.63 ^f^
	T = 150 °C, RH = 0.3%	1475.61 ± 1.75 ^h^	903.34 ± 1.68 ^d^	987.45 ± 1.55 ^e^
	T = 150 °C, RH = 5.0%	1411.21 ± 1.62 ^g^	897.71 ± 1.64 ^d^	904.74 ± 1.49 ^d^
**Forastero** (Ghana)	unroasted	689.16 ± 1.35 ^a^	806.22 ± 1.67 ^a^	553.90 ± 1.49 ^a^
	T = 110 °C, RH = 0.3%	1046.65 ± 1.86 ^d^	917.64 ± 1.59 ^d,e^	785.85 ± 1.52 ^d^
	T = 110 °C, RH = 5.0%	938.30 ± 1.88 ^b^	857.05 ± 1.78 ^b^	714.41 ± 1.68 ^b^
	T = 120 °C, RH = 0.3%	1124.59 ± 1.79 ^e^	937.50 ± 1.72 ^e^	825.15 ± 1.71 ^e^
	T = 120 °C, RH = 5.0%	989.95 ± 1.85 ^c^	903.18 ± 1.65 ^d^	750.13 ± 1.63 ^c^
	T = 135 °C, RH = 0.3%	1224.05 ± 1.89 ^g^	819.04 ± 1.68 ^a^	947.46 ± 1.70 ^h^
	T = 135 °C, RH = 5.0%	1172.50 ± 1.67 ^f^	826.16 ± 1.57 ^a^	911.02 ± 1.56 ^g^
	T = 150 °C, RH = 0.3%	1309.52 ± 1.91 ^h^	897.62 ± 1.64 ^c,d^	817.99 ± 1.59 ^e^
	T = 150 °C, RH = 5.0%	1177.01 ± 1.84 ^f^	877.13 ± 1.71 ^b,c^	850.71 ± 1.58 ^f^
**Trinitario** (Madagascar)	unroasted	640.43 ± 1.29 ^a^	680.90 ± 1.56 ^a^	440.49 ± 1.42 ^a^
	T = 110 °C, RH = 0.3%	1024.17 ± 1.45 ^d^	833.81 ± 1.71 ^c^	728.69 ± 1.71 ^g^
	T = 110 °C, RH = 5.0%	1006.96 ± 1.37 ^d^	874.36 ± 1.39 ^d^	698.13 ± 1.64 ^e^
	T = 120 °C, RH = 0.3%	1209.25 ± 1.39 ^f^	976.25 ± 1.48 ^g^	624.41 ± 1.72 ^c^
	T = 120 °C, RH = 5.0%	1107.30 ± 1.27 ^e^	948.24 ± 1.53 ^f^	590.76 ± 1.59 ^b^
	T = 135 °C, RH = 0.3%	938.00 ± 1.68 ^c^	897.84 ± 1.67 ^e^	779.49 ± 1.76 ^i^
	T = 135 °C, RH = 5.0%	834.00 ± 1.46 ^b^	894.13 ± 1.56 ^d,e^	768.42 ± 1.36 ^h^
	T = 150 °C, RH = 0.3%	1189.09 ± 1.24 ^f^	714.99 ± 1.41 ^b^	654.89 ± 1.52 ^d^
	T = 150 °C, RH = 5.0%	1085.65 ± 1.37 ^e^	689.24 ± 1.72 ^a^	584.27 ± 1.65 ^b^
**Two-way ANOVA analysis**		Significance		
Variety [V]		**	*	***
Roasting temperature [RT]		***	***	***
Roasting air humidity [RH]		**	ns	**
Interactions of V × RT × RH		***	***	***

T, temperature. RH, relative air humidities. Data are presented as mean ± SD of three replications. The means followed by the same lowercase letter (^a–h^) within each variety in the same column do not differ significantly according to Tukey’s HSD test at *p* < 0.05. Significance: * *p* < 0.05; ** *p* < 0.01; *** *p* < 0.001; ns: not significant. DPPH, the DPPH radical-scavenging capacity expressed in µmol Trolox equivalents per gram of HMW fraction dry weight (μmol TE/g DW); ABTS, the ABTS radical-scavenging activity expressed in in µmol Trolox equivalents per gram of HMW fraction dry weight (μmol TE/g DW). FRAP, the ferric reducing antioxidant power expressed in µmol Trolox equivalents per gram of HMW fraction dry weight (μmol TE/g DW).
